# Functional metabolic interactions of human neuron-astrocyte 3D *in vitro* networks

**DOI:** 10.1038/srep33285

**Published:** 2016-09-13

**Authors:** Daniel Simão, Ana P. Terrasso, Ana P. Teixeira, Catarina Brito, Ursula Sonnewald, Paula M. Alves

**Affiliations:** 1 iBET, Instituto de Biologia Experimental e Biológica, Oeiras, Portugal; 2 Instituto de Tecnologia Química e Biológica António Xavier, Universidade Nova de Lisboa, Oeiras, Portugal; 3 Department of Neuroscience, Norwegian University of Science and Technology (NTNU), Trondheim, Norway; 4 Department of Drug Design and Pharmacology, Faculty of Health and Medical Sciences, University of Copenhagen, Copenhagen, 2100, Denmark

## Abstract

The generation of human neural tissue-like 3D structures holds great promise for disease modeling, drug discovery and regenerative medicine strategies. Promoting the establishment of complex cell-cell interactions, 3D culture systems enable the development of human cell-based models with increased physiological relevance, over monolayer cultures. Here, we demonstrate the establishment of neuronal and astrocytic metabolic signatures and shuttles in a human 3D neural cell model, namely the glutamine-glutamate-GABA shuttle. This was indicated by labeling of neuronal GABA following incubation with the glia-specific substrate [2-^13^C]acetate, which decreased by methionine sulfoximine-induced inhibition of the glial enzyme glutamine synthetase. Cell metabolic specialization was further demonstrated by higher pyruvate carboxylase-derived labeling in glutamine than in glutamate, indicating its activity in astrocytes and not in neurons. Exposure to the neurotoxin acrylamide resulted in intracellular accumulation of glutamate and decreased GABA synthesis. These results suggest an acrylamide-induced impairment of neuronal synaptic vesicle trafficking and imbalanced glutamine-glutamate-GABA cycle, due to loss of cell-cell contacts at synaptic sites. This work demonstrates, for the first time to our knowledge, that neural differentiation of human cells in a 3D setting recapitulates neuronal-astrocytic metabolic interactions, highlighting the relevance of these models for toxicology and better understanding the crosstalk between human neural cells.

There has been an increasing demand for a paradigm shift from animal cells towards robust human cell models of high physiological relevance^1^. This is due to the fact that over the last years, the drug discovery and development pipelines have registered low success rates both during preclinical and clinical phases, with only 23 small molecules and 2 biologics being approved by the Food and Drug Administration (FDA) in 2013^2^,^3^. These is even more dramatic for neurological disorders, such as Alzheimer’s disease for which there is an estimated overall success rate of only 0.5%^4^. Although several factors have been described to contribute to this, the current lack of adequate and predictive preclinical models plays an important role. Most of the available preclinical models are based on animals or immortalized cell lines, which do not accurately recapitulate key events of human pathologies^5^,^6^,^7^. In this context, the generation of human neural 3D cell models holds great promise for drug discovery, toxicology and disease modeling.

Multiple human neural cell sources have become available, including immortalized cell lines, embryonic or adult stem cells and induced pluripotent stem cells (iPSC)^8^. The human Ntera2/clone D1 (NT2) cell line has been extensively characterized, demonstrating its ability to generate neuronal and astrocytic cells^9^,^10^,^11^. NT2 neural differentiation has been shown to recapitulate developmental processes observed during *in vivo* neurogenesis^12^, generating neuronal cells able to establish functional synapses and elicit action potentials following depolarization^13^,^14^. Given these properties, the NT2 cell line has been reported to be a promising human cell source for toxicological applications^15^,^16^,^17^,^18^. Traditional cell culture systems based on cell monolayers do not reflect the *in vivo* architecture complexity, mechanical and biochemical cues, failing to mimic important features of the target tissue/organ. Conversely, 3D culture systems allow to recapitulate the *in vivo* cell-cell and cell-extracellular matrix interactions (ECM), while presenting an intermediate degree of complexity between traditional 2D cultures and the organ^19^. Different strategies can be explored for 3D cell culture, including embedding cells in artificial matrices/scaffolds^20^ or as cell aggregates^21^. Neural differentiation of human stem cells in these 3D systems results in a heterogeneous culture, composed of the different neural cell lineages present in the brain: neurons, astrocytes and oligodendrocytes^15^,^22^,^23^. Complex cell-cell interactions and networks can be established within these cell aggregates, or neurospheres, making it possible to mimic key brain features, such as synaptic activity or electrophysiological properties. Recently, Choi *et al*. have been able to recapitulate amyloid-β and tau pathological events of Alzheimer’s disease in a human 3D cell model, which was not possible to model in rodents nor in 2D cultures^24^. Human neural 3D cell models can thus be valuable tools to study human neural metabolism^25^, as this field has traditionally relied on rodent models, using both *in vivo* studies and primary cultures, which can diverge considerably from the human phenotype and make it impossible to develop appropriate treatment approaches.

The brain’s homeostasis depends on multiple metabolic cycles between the different cell populations that represent major hallmarks of neural metabolism. For instance, as neurons are continually loosing tricarboxylic acid (TCA) cycle intermediates, mainly for the synthesis of neurotransmitters (α-ketoglutarate is converted into glutamate and subsequently GABA), compensatory mechanisms have to act in order to replenish the carbon skeleton for maintaining TCA cycle activity^26^. Some metabolic neural features have been showed to be present in 2D cultures of stem cell-derived neural cells, as glycogen synthesis in astrocytes or the ability to modulate glucose consumption in response to glutamate addition^27^. The presence of astrocytes in co-cultures with neurons has been shown also to contribute to a closer recapitulation of *in vivo* features, such as the dopaminergic neurodegeneration process following administration of neurotoxicants^28^,^29^. *In vitro* co-culture systems have also been proposed to be relevant tools for studying neurodevelopmental processes, including neuritogenesis and synaptogenesis^30^,^31^. Still, other important neural metabolic hallmarks, including the glutamine-glutamate-GABA cycle between neurons and astrocytes^32^ have not been previously reported for these stem-cell derived co-cultures cultures. This specific neuron-astrocyte shuttle is one of the major metabolic specialization and compartmentation in brain tissue, where astrocytes are able to take up glutamate or GABA released by neurons into the synaptic cleft and convert glutamate to glutamine or GABA to Succinyl CoA, entering the TCA cycle via the GABA shunt. Astrocyte derived-glutamine is then transported back to neurons, acting as precursor for neurotransmitter synthesis. These cycles are critical in order to control the excitotoxic effects of glutamate as well as to feed neurons with a neurotransmitter precursor.

The use of ^13^C-labeled substrates and nuclear magnetic resonance (NMR) spectroscopy are powerful tools for the study of neural metabolic networks, having provided important insights into the biochemical mechanisms and compartmentation of neural metabolism^33^,^34^,^35^. Different ^13^C-labeled substrates have been used to study neuronal-glial metabolic interactions, taking advantage of cell specific metabolic features. An example of this metabolic specialization is the fact that glucose is taken up mostly by neurons^36^, while astrocytes have been shown to metabolize acetate^37^,^38^.

In this work, we combined the use of a human 3D *in vitro* neural model with ^13^C-NMR spectroscopy to study the metabolic features of human neuronal-astrocytic networks. Furthermore, to study neuronal-glial metabolic interactions in detail the cells were challenged with compounds targeting specifically astrocytic or neuronal populations, using methionine sulfoximine (MSO) and acrylamide, respectively. This study demonstrates that some of the main metabolic features and specializations found in brain tissue are recapitulated in human neuron-astrocyte 3D *in vitro* networks.

## Materials and Methods

### Cell culture and 3D neural differentiation

Ntera-2 (NT2) cells were routinely cultivated in standard tissue culture flasks (Sarstedt) and maintained in DMEM (Life Technologies) supplemented with 10% (v/v) fetal bovine serum (FBS; Hyclone) and 1% (v/v) penicillin-streptomycin (P/S; Life Technologies), as previously described^39^. NT2 neural differentiation was performed as previously described^15^. Briefly, 125 mL silanized spinner flasks (Wheaton) equipped with ball impeller were inoculated with a single cell suspension at 6.7 × 10^5^ cell/mL in 75 mL of DMEM with 10% FBS and 1% P/S. On the day after, 50 mL of fresh medium were added. At day 3, neural differentiation was induced adding 20 μM retinoic acid (RA; Sigma-Aldrich), by performing a 50% medium exchange. RA treatments were performed every 2–3 days, during 3 weeks. After this period, cells were posteriorly maintained in DMEM with 5% (v/v) FBS, 1% (v/v) P/S and absence of RA during 2 weeks, for neuronal maturation. Spinner flasks stirring speed was gradually increased from 40 to 100 rpm throughout culture time. Cells were maintained in a humidified atmosphere of 5% CO_2_ in air at 37 °C.

### Incubation with ^13^C labeled substrates

After neural differentiation process (day 38), two 50% media exchanges were performed in the following two days of culture with a low glucose DMEM medium (5.5 mM glucose), 5% FBS and 1% P/S. At day 3 the medium was completely removed and replaced by 100 mL of: (i) DMEM with 5.5 mM [1-^13^C]glucose, 3 mM acetate and 5% FBS or (ii) DMEM with 5.5 mM glucose, 3 mM [2-^13^C]acetate and 5% FBS. After 12 hours incubation, neurospheres were harvested, centrifuged at 300 × *g* for 2 minutes and washed 2 times in ice-cold PBS followed by centrifugation. The obtained cell pellets were the immersed in liquid nitrogen and 2 mL of 70% (v/v) ethanol was added. Complete cell lysis was achieved by ultrasound sonication (Sonifier 250D, Branson). The cell extracts were centrifuged two times at 20,000 × *g* for 15 minutes. Cell pellets were stored at −80 °C for total protein quantification.

### Fate of [1-^13^C]glucose and [2-^13^C]acetate

**Figure 1:**
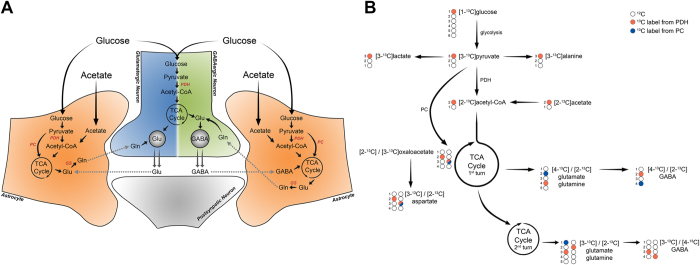
(**A**) Schematic representation outlining the main neuronal-astrocytic metabolic networks and glutamine-glutamate/GABA cycles. (**B**) ^13^C-labeling patterns of metabolites derived from [1-^13^C]glucose and [2-^13^C]acetate. ^13^C-label can enter the TCA clycle via pyruvate dehydrogenase (PDH; orange circle) or via pyruvate carboxylase (PC; blue circle).

A simplified representation of the main neuronal and astrocytic metabolic networks, as well as the labeling patterns resulting from the metabolism of [1-^13^C]glucose and [2-^13^C]acetate, is shown in Fig. 1. Through glycolysis, [1-^13^C]glucose can be metabolized into [3-^13^C]pyruvate, which can then be reduced to [3-^13^C]lactate or transaminated to [3-^13^C]alanine. The labeled pyruvate molecules can enter the tricaboxylic acid (TCA) cycle in the mitochondria via pyruvate dehydrogenase (PDH) with formation of [2-^13^C]acetyl-CoA, which is converted to [2-^13^C]citrate. With the progression of the TCA cycle, α-[4-^13^C]ketoglutarate is formed and can exit the cycle for the synthesis of [4-^13^C]glutamate. This can act as neurotransmitter, in case of glutamatergic neurons, or be used to fuel the synthesis of [4-^13^C]glutamine in astrocytes or [2-^13^C]GABA in GABAergic neurons. In case α-[4-^13^C]ketoglutarate remains in the TCA cycle, the label will be scrambled by the formation of succinate that is a symmetric molecule, resulting in equal amounts of [2-^13^C]- and [3-^13^C]succinate that gives rise to fumarate, malate and oxaloacetate labeled in the same positions. [2-^13^C]- and [3-^13^C]oxaloacetate can also exit the cycle by being converted into aspartate, which will have the same carbon positions labeled. If the cycle continues with the labeled oxaloacetate, this will be condensed with an acetyl-CoA molecule, which can be unlabeled or labeled. In presence of unlabeled acetyl-CoA, [3-^13^C]- or [2-^13^C]glutamate/glutamine and [3-^13^C]- or [4-^13^C]GABA will be generated from the second turn of TCA cycle. In case the of labeled acetyl-CoA, the generated molecules will also present labeling in the positions described previously for the first turn, resulting in [3,4-^13^C]- or [2,4-^13^C]glutamate/glutamine and [2,3-^13^C]- or [2,4-^13^C]GABA.

Alternatively, [3-^13^C]pyruvate can enter the TCA cycle through the anaplerotic pathway, which in neural cells is achieved via pyruvate carboxylase (PC), leading to the formation of [3-^13^C]oxaloacetate. This can exit the cycle by transamination generating [3-^13^C]aspartate or proceed in the cycle forming α-[2-^13^C]ketoglutarate, which can act as precursor for the synthesis of [2-^13^C]glutamate/glutamine and [4-^13^C]GABA. In case the label remains in the cycle for a second turn, [1-^13^C]glutamate/glutamine will be formed and the decarboxylation of glutamate into GABA will result in the loss of the labeled carbon. It should be noted that backflux of labeled oxaloacetate after carboxylation of pyruvate will lead to scrambling of the label from the C3 position in oxaloacetate to the C2 position and make it indistinguishable from oxaloacetate obtained from cycling. Thus, pyruvate carboxylation assessment using [1-^13^C]glucose can be underestimated, since backflux is not taken into account^40^.

Incubation with [2-^13^C]acetate results in its conversion into [2-^13^C]acetyl-CoA, which will then enter the TCA cycle and lead to a labeling pattern similar to what was described above for glucose.

### Incubation with toxic compounds

For the experiments with toxic compounds, two 50% media exchanges with low glucose medium were also performed in two consecutive days after neural differentiation (day 38). For acrylamide incubation experiments, at day 3 after differentiation, the medium was completely removed and replaced by DMEM with 5.5 mM glucose, 3 mM acetate, 2.5 mM acrylamide and 5% FBS. After 36 hours of incubation, the medium was completely exchanged with a medium of similar composition but with [1-^13^C]glucose and maintained for further 6 hours. For methionine sulphoximine (MSO) experiments, at day 3 after differentiation, the medium was completely removed and maintained for 12 hours with DMEM containing 5.5 mM glucose, 3 mM [2-^13^C]acetate, 20 μM MSO and 5% FBS. Following incubation with labeled substrates, cells were harvested as described above. Control experiments were performed in parallel under the same experimental conditions except for the absence of the toxic compounds.

### NMR sample preparation and analysis

Lyophilized samples were prepared as previously decribed^41^, by dissolving in 120 mL D_2_O containing 0.25% ethylene glycol and 0.002% TSP. Samples were then transferred to SampleJet tubes (3.0 ± 103.5 mm). All samples were analyzed on a 14.1T Ultra shielded Plus Avance III magnet (Bruker BioSpin GmbH, Rheinstetten, Germany) operating at 600 MHz (for 1H) using QCI CryoProbe (Bruker BioSpin GmbH) and equipped with SampleJet auto sampler (Bruker BioSpin GmbH). ^1^H-NMR spectra were accumulated with a pulse angle of 90°, 2.7 s acquisition time, and 10 s relaxation delay. The number of scans was 128. Proton decoupled ^13^C-NMR spectra were obtained using an acquisition time of 1.7 s, 0.5 s relaxation delay, and a 30° flip angle. Scans were accumulated at 30 kHz spectral width with 98 K data points. The number of scans was typically 2000. Relevant peaks in the spectra were identified and integrated using MNova software (Mestrelab Research, Santiago de Compostela, Spain). Concentrations of metabolites were calculated from the integrals of the peaks using TSP (^1^H-NMR spectra) or ethylene glycol (^13^C-NMR spectra) as internal quantification standards. Concentrations from the ^1^H-NMR spectra were corrected for the numbers of protons that constituted the peak. Correction factors for incomplete relaxation and nuclear Overhauser effects in the ^13^C spectra were obtained by acquiring scans with completely relaxed nuclei (relaxation delay of 20 s) and only a brief proton decoupling during the radiofrequency pulse shortly before the signal was recorded to avoid heteronuclear splitting of signals, but no proton decoupling for the remainder of the acquisition to avoid nuclear Overhauser effects. The singlets in the ^13^C-nuclear magnetic resonance spectroscopy (^13^C-NMRS) data were corrected for naturally abundant ^13^C by subtracting 1.1% of the total cellular contents obtained from ^1^H spectra or HPLC data. All amounts were corrected for total protein weight. Percent excess enrichment was calculated after subtracting natural abundance where appropriate, and is in the following referred to as percent enrichment.

### HPLC analysis

Amino acids were quantified by high performance liquid chromatography (HPLC) using a pre-column derivatization method based on the Waters AccQ. Tag Amino Acid Analysis (Waters, USA) as previously described^42^. Briefly, sample proteins were precipitated by adding an equal volume of acetonitrile and removed by centrifugation at 13,000 × *g* for 15 minutes. The obtained supernatants were used for primary and secondary aminoacid derivatization by mixing with 6-aminoquinolyl N-hydroxysuccinimidyl-carbamate, which allows their separation in a reversed phase column (Waters, USA) and the detection of fluorescence at 395 nm. As internal standard, α-aminobutyric acid was added to ensure consistency between runs. Mobile phases were prepared following the manufacturer’s instructions, filtered and degassed in an ultrasound bath before usage.

### Total protein quantification

Total cell biomass was evaluated by quantification of total protein using the bicinchoninic acid (BCA) protein assay kit (Pierce), after dissolving the cell pellet in Tris buffer (50 mM Tris, 5 mM EDTA, 150 mM NaCl, pH 7.4).

### Immunofluorescence microscopy

Neurospheres were fixed in 4% (w/v) paraformaldehyde (PFA) + 4% (w/v) sucrose in PBS for 20 min and processed for immunostaining as previously described^43^. Primary and secondary antibodies were used as follows: mouse anti-βIII-tubulin (1:200; Millipore Darmstadt, Germany, MAB1637); rabbit anti-GFAP (1:200; Millipore, AB5804); AlexaFluor^®^ 488 goat anti-mouse IgG (1:500; Life Technologies, A11001) and AlexaFluor^®^ 594 goat anti-rabbit IgG (1:500; Life Technologies, A11012). Cell nuclei were counterstained with TO-PRO-3 (Life Technologies). Samples were visualized using point-scan confocal microscopy (SP5, Leica, Wetzlar, Germany) or light-sheet microscopy^44^,^45^. Merge between channels and maximum z-projections, as well as linear brightness and contrast adjustments of the images, were performed using the open source FIJI software^46^.

### Real time RT-PCR

Total RNA was extracted with High Pure RNA Isolation Kit (Roche), according to the manufacturer’s instructions. RNA was quantified in a NanoDrop 2000c (Thermo Scientific) and used for cDNA synthesis. Reverse transcription was performed with High Fidelity cDNA Synthesis Kit (Roche), using Anchored-oligo(dT)18 Primer (Roche) or with the Super Script III First Strand synthesis system (Invitrogen), using random hexamers (Invitrogen). qPCRs were performed in triplicates using LightCycler 480 SYBR Green I Master Kit (Roche) with the following primers: βIII-tubulin (*TUBB3;* fwd 5′-GGGCCTTTGGACATCTCTTC-3′ and rev 5′-CCTCCGTGTAGTGACCCTTG-3′), glial fibrillary acidic protein (*GFAP*; fwd 5′-AGAGAGGTCAAGCCCAGGAG-3′ and rev 5′-GGTCACCCACAACCCCTACT-3′) and ribosomal protein L22 (*RPL22*; fwd 5′-CACGAAGGAGGAGTGACTGG-3′ and rev 5′-TGTGGCACACCACTGACATT-3′). The reactions were performed with LightCycler 480 Instrument II 96-well block (Roche). Quantification cycle values (C_q_’s) and melting curves were determined using LightCycler 480 Software version 1.5 (Roche). All data were analyzed using the 2^−ΔΔCt^ method for relative gene expression analysis^47^. Changes in gene expression were normalized using the housekeeping gene *RPL22* as internal control.

### Synaptic activity assessment

Neurospheres plated on poly-D-lysine (PDL)-coated multi-well plates were washed with PBS prior to a 5 minutes incubation with 100 mM KCl Buffer (5 mM HEPES-NaOH, pH 7.4; 10 mM glucose; 2.5 mM calcium chloride; 1 mM magnesium chloride; 37 mM sodium chloride; 100 mM potassium chloride). Afterwards, neurospheres were incubated for 15 minutes with 10 mM FM1–43 dye (Life Technologies) dissolved in normal saline buffer (5 mM HEPES-NaOH, pH 7.4; 10 mM glucose; 2.5 mM calcium chloride; 1 mM magnesium chloride; 37 mM sodium chloride; 5 mM potassium chloride). Neurospheres were then washed with ADVASEP-7 (Sigma) dissolved in 5 mM KCl buffer for 1 min, followed by three washes of 1 min with 5 mM KCl buffer. Exocytosis was stimulated with 100 mM KCl buffer and samples were imaged live in a fluorescence microscope (DMI6000; Leica) to monitor synaptic vesicle release. Fluorescence intensity was measured using the open source FIJI software^46^. FM1-43 unloading analyses were performed after normalization by dividing the fluorescence intensity of each frame by the initial fluorescence intensity. FM1-43 fluorescence decrease rate was determined by a linear regression analysis of the normalized fluorescence intensity time profile for the 15 minutes following KCl stimuli.

### Statistical Analysis

All values are presented as means ± standard error of the mean (s.e.m.). All analyticals were performed as triplicates from samples of two independent experiments. Student’s t-tests were used to compare means. Before choosing the adequate type of t-test, Levene’s test for equal variances was performed. P < 0.05 was chosen as the level of significance. All comparisons were made using two-tailed statistical tests.

## Results

### Neuronal and glial metabolic signatures and trafficking

**Figure 2:**
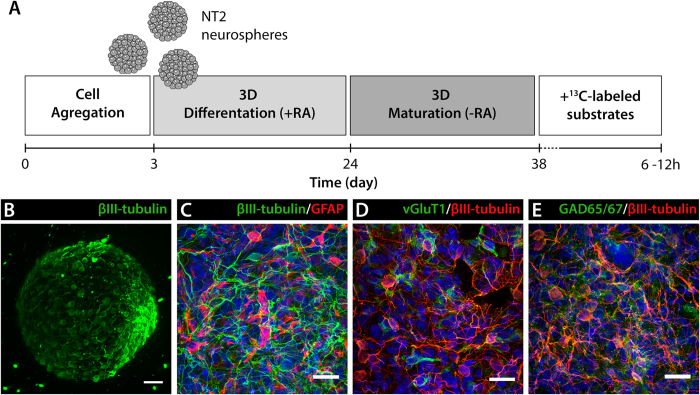
(**A**) Schematic experimental workflow for NT2 3D differentiation. Cells were inoculated in a stirred suspension culture system and aggregated for 3 days. Neural differentiation was induced by exposure to retinoic acid (RA) for 3 weeks, followed by 2 weeks without RA. Differentiated neurospheres were further cultured in presence of ^13^C-labeled substrates for 6-12 hours. (**B**) Immunofluorescence light-sheet microscopy of neurospheres stained for βIII-tubulin. Scale bar, 50 μm. (**C–E**) Immunofluorescence light-sheet microscopy of neurospheres stained for βIII-tubulin and GFAP (**C**), βIII-tubulin and vGluT1 **(D)**, βIII-tubulin and GAD65/67 (**E**). Scale bars, 20 μm.

NT2 aggregates differentiation towards the neural lineage can be achieved upon RA induction and subsequent maturation (Fig. 2A), resulting in highly viable (Fig. 2B) differentiated neurospheres composed mainly of βIII-tubulin-positive neurons and GFAP-positive astrocytes (Fig. 2C), as previously reported by our group^15^. Phenotypic characterization demonstrated that the neuronal population was comprised by glutamatergic (vGluT1-positive) and GABAergic (GAD65/67-positive) neurons (Fig. 2D,E). In this study, differentiated NT2 neurospheres were incubated with [1-^13^C]glucose or [2-^13^C]acetate, and the fate of the ^13^C label was observed by ^13^C-NMR spectroscopy for the identification of major neural metabolic signatures.

**Figure 3:**
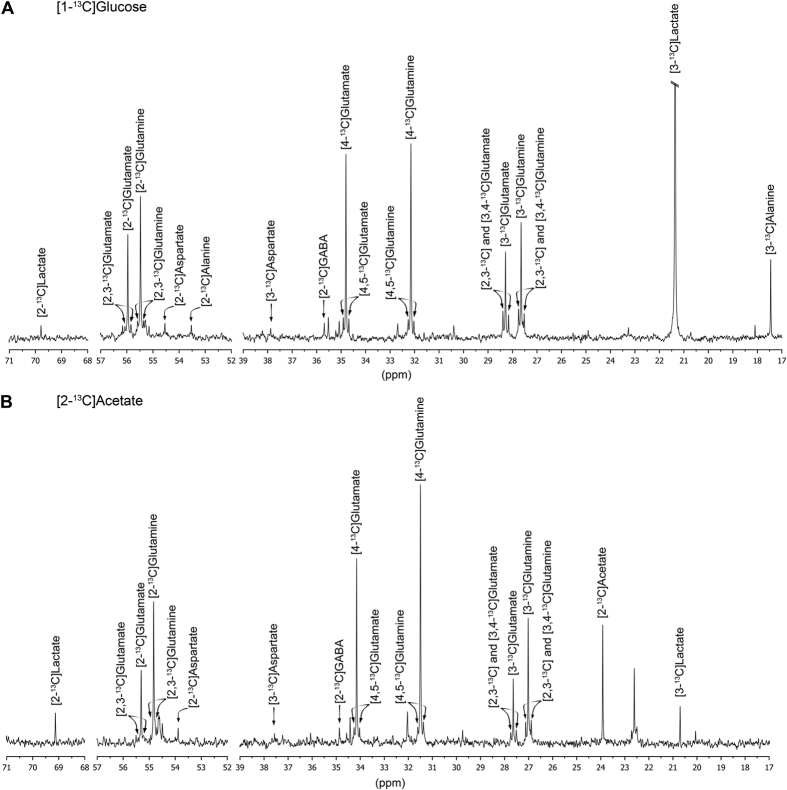
Typical ^13^C-NMR spectra of cellular extracts from neurospheres incubated with [1-^13^C]glucose (**A**) or [2-^13^C]acetate (**B**). Relevant metabolites are identified.

**Figure 4:**
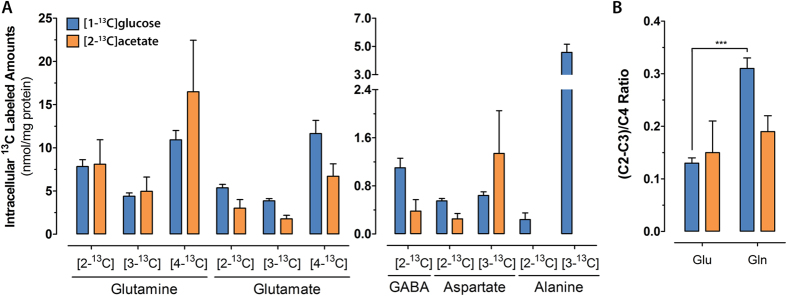
(**A**) Intracellular amounts of ^13^C labeled metabolites in cultures incubated with [1-^13^C]glucose (blue bars) or [2-^13^C]acetate (orange bars). (**B**) Metabolic ratios for PC/PDH described by the (C2-C3)/C4 ratio of labeled glutamate (Glu) and glutamine (Gln) in cultures incubated with [1-^13^C]glucose (blue bars) or [2-^13^C]acetate (orange bars). Data are mean ± s.e.m. P values are given for PC/PDH ratio (analysis of glutamate versus glutamine): ***P < 0.001. PC, pyruvate carboxylase; PDH, pyruvate dehydrogenase.

Typical ^13^C-NMR spectra after incubation with each ^13^C labeled substrate are presented in Fig. 3. Incubation with [1-^13^C]glucose resulted in detectable levels of labeled glutamine, glutamate, lactate, alanine, aspartate and GABA (Fig. 4A and Table 1). After 12 hours of incubation, glutamate and glutamine were mainly labeled at C4, which results from one turn in the TCA cycle (Fig. 1). Additionally, the presence of labeling in C2 and C3 positions indicates label cycling in the TCA cycle. The amounts of [2-^13^C]glutamine were higher relative to [2-^13^C]glutamate, while the labeling in C3 was similar for both. This reflects the different contributions of the anaplerotic (via PC) and oxidative (via PDH) pathways for the synthesis of these two metabolites. These contributions can be expressed as the ratio PC/PDH, which can be estimated by dividing the difference between C2 and C3 by C4 ((C2-C3)/C4)^48^. This was found to be 2.3-fold higher for glutamine in comparison with glutamate (Fig. 4B), revealing an increased contribution of PC over PDH for glutamine synthesis. GABA labeling was only observed in the C2 position, which derives from the decarboxylation of the most abundant glutamate isotopomer (C4). Aspartate was labeled in the C2 and C3 positions with no significant differences between the two. Labeling of alanine and lactate in the C3 position was also observed, which derive from [3-^13^C]pyruvate molecules generated from glycolysis.

Incubation with [2-^13^C]acetate during 12 hours resulted also in significant labeling of glutamate, glutamine, aspartate and GABA (Fig. 4A and Table 1). The percentage of ^13^C enrichment was similar or slightly lower than that from [1-^13^C]glucose in all metabolites, except in glutamine, where a higher ^13^C incorporation from [2-^13^C]acetate is in accordance with acetate being a major carbon source for glutamine synthesis in astrocytes. As observed with [1-^13^C]glucose, labeling in C4 was the most common in glutamate and glutamine. Moreover, significant differences were not observed for the PC/PDH ratio for glutamine and glutamate, as in this case there is not contribution from the PC pathway for the labeling pattern, since [2-^13^C]acetate is converted into [2-^13^C]acetyl-CoA that enters directly the TCA cycle (Fig. 4B). Finally, labeling of GABA in the C2 position was observed (Figs 3 and 4A), suggesting the trafficking of labeled glutamine from astrocytes to neurons that acts as precursor for GABA synthesis.

### Inhibition of astrocytic glutamine synthesis

To isolate the contribution of astrocytic-derived glutamine as precursor for neuronal neurotransmitter synthesis, following the glutamine-glutamate/GABA shuttles between neurons and astrocytes, differentiated neurospheres were incubated with MSO, a specific glutamine synthase (GS) inhibitor.

**Figure 5:**
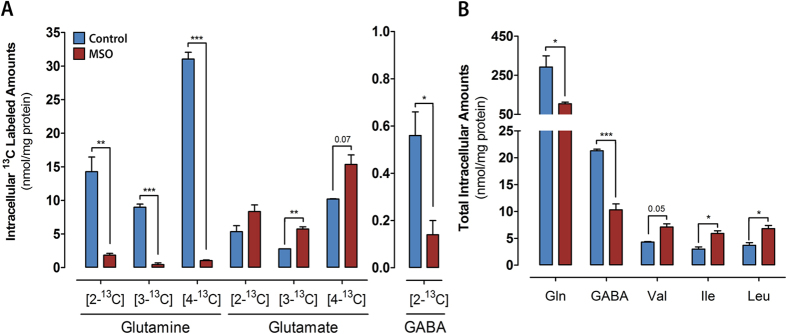
(**A**) Intracellular amounts of ^13^C labeled metabolites in cultures incubated with [2-^13^C]acetate alone (blue bars) or [2-^13^C]acetate plus MSO (red bars). (**B**) Total concentration of intracellular metabolites in control (blue bars) and MSO-treated cultures (red bars). Data are mean ± s.e.m. Asterisks indicate significant difference: *P < 0.05; **P < 0.01; ***P < 0.001; near significant *P* values (<0.1) are given in numbers. Gln, glutamine; Val, valine; Ile, isoleucine; Leu, Leucine.

The presence of MSO in the culture medium resulted in an almost complete absence of ^13^C incorporation in glutamine coming from [2-^13^C]acetate (Fig. 5A) and a 2.8-fold decrease in the intracellular glutamine pool (Fig. 5B). In accordance with the role of astrocytic glutamine as a key precursor for GABA synthesis in neurons, decreased [2-^13^C]GABA labeling and a 50% reduction in intracellular GABA content was also observed (Fig. 5A,B). As for glutamate, its total intracellular pool was not affected in MSO-treated cultures. Nevertheless, a modest increase in ^13^C labeled glutamate was observed, mostly visible in the amount of [3-^13^C]glutamate, suggesting a slower degradation or accumulation, as its condensation with ammonia to generate glutamine is impaired by MSO. The catabolism of branched chain amino acids (BCAA), comprising valine, isoleucine and leucine, was decreased in cultures exposed to MSO, as shown by the significantly higher intracellular pools detected (Fig. 5B).

### Impairment of neuronal metabolism

**Figure 6:**
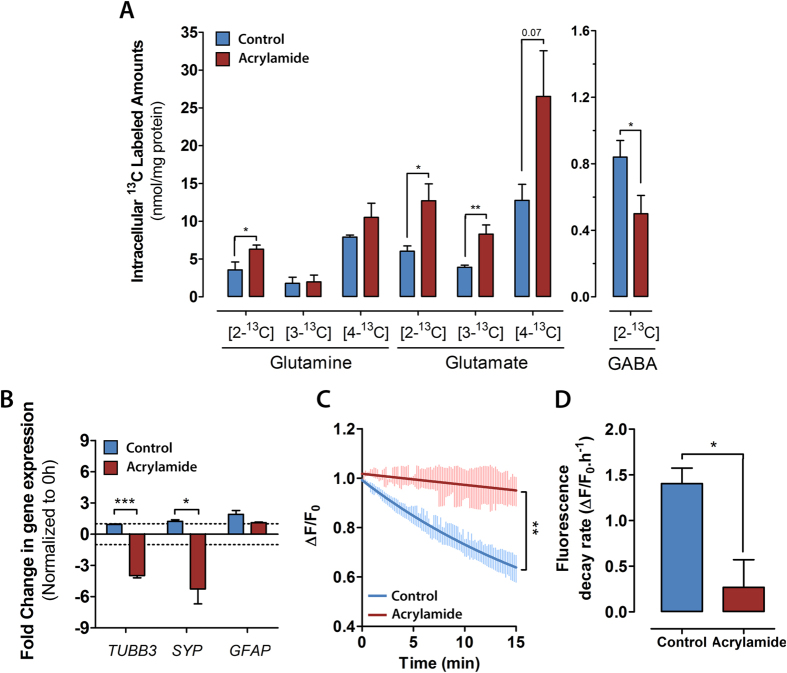
(**A**) Intracellular amounts of ^13^C labeled metabolites in cultures incubated with [1-^13^C]glucose alone (blue bars) and [1-^13^C]glucose plus acrylamide (red bars). (**B**) Gene expression analysis of differentiated neurospheres in control (blue bars) and acrylamide-treated cultures (red bars). Gene expression fold changes (normalized to expression levels previous to incubation) of neuronal markers (*TUBB3*, *SYP*) and glial marker (*GFAP*). (**C**) Synaptic activity assessment by FM1-43 unloading of differentiated neurospheres in control (blue trace) and acrylamide-treated cultures (red trace). Data represents a single exponential fit and standard deviation of the measured fluorescence values. (**D**) FM1-43 fluorescence decrease rate determined for control (blue bar) and acrylamide-treated cultures (red bar). Data are mean ± s.e.m. Asterisks indicate significant difference: *P < 0.05; **P < 0.01; ***P < 0.001; near significant *P* values (<0.1) are given in numbers.

The NT2 neural cells were further challenged by incubating the neurospheres with [1-^13^C]glucose in the presence of acrylamide, a toxic compound that specifically targets the neuronal population. The specificity of acrylamide toxicity towards neurons was confirmed by gene expression analysis of neuronal (*TUBB3* and *SYP*) and glial (*GFAP*) specific transcripts. After incubation with acrylamide, *TUBB3* and *SYP* expression levels were significantly lower in comparison with control, with a 4- and 5.3-fold decrease respectively, while no modulation on *GFAP* expression was observed (Fig. 6B).

^13^C-NMRS analysis revealed significant changes in the amounts of labeled glutamate and GABA after incubation with acrylamide (Fig. 6A). While the amounts of labeled glutamate in the C2, C3 and C4 positions were significantly higher in comparison with the control, [2-^13^C]GABA amounts significantly decreased in the presence of acrylamide. Additionally, a significant increase of [2-^13^C]glutamine was observed, suggesting an increased contribution of the anaplerotic pathway for glutamine synthesis upon acrylamide exposure. As for the total intracellular metabolite pools, no significant differences were detected between acrylamide exposed neurospheres and control (data not shown).

The effects of acrylamide on neuronal functionality were also assessed. For this, synaptic activity of differentiated neurospheres was evaluated using the fluorescent probe FM1-43, which provides a direct measurement of synaptic vesicle release following a depolarizing KCl stimulus^49^,^50^. The analysis of FM1-43 unloading kinetics demonstrated that the same stimuli induced a lower dimming of fluorescence intensity in acrylamide-treated neurospheres (Fig. 6C), which was reflected by a significantly lower fluorescence decrease rate in comparison with control neurospheres (Fig. 6D).

## Discussion

We have previously demonstrated that 3D cultures of human mature neurons and functional astrocytes can be derived from NT2 cells^15^. The use of this 3D differentiation protocol was also shown to attain higher cell yields in half of the culture time, as compared with 2D cultures^16^,^43^. Importantly, NT2 differentiated neurospheres present a cellular organization that better mimics the *in vivo* tissue structural features, with an interspersed distribution of neurons and astrocytes. In 2D cultures, these neuron-glia interfaces are much less pronounced, resulting in a compartmentalized cellular organization where neurons bundle in small aggregates on top of a monolayer of glial cells^51^,^52^. Recent reports support the increased inter-cellular communications established in 3D neural models via secreted mediators, as the extracellular deposition of amyloid-β that was only observed in 3D cultures of human differentiated neurons expressing familial Alzheimer’s disease (FAD) mutations^24^; neurodevelopment regional specification^53^ and the effects of viral pathogen ZIKV exposure during neurodevelopment^54^. Therefore, metabolic data from 3D neural cell models can provide valuable insights, by offering the possibility to better understand the biochemical changes induced by compounds of interest in a controlled setting^25^. In this context, the metabolic specialization and establishment of functional metabolic shuttles between the different cell compartments are major hallmarks of brain functionality. Thus, the main goal of this work was to investigate whether human neural cells generated in 3D cultures were able to acquire the main metabolic features and interactions found in the human brain.

To analyze the metabolic profile of the differentiated NT2 neurospheres, we followed the fate of two different ^13^C-labelled substrates: [1-^13^C]glucose and [2-^13^C]acetate. While glucose acts as carbon source mainly for neurons, acetate is only taken up and metabolized by astrocytes^37^,^38^. Using both substrates it is possible to assess the presence of the two different cellular compartments by following the incorporation of the label by ^13^C NMR spectroscopy. This approach had previously been explored by our group for deciphering the main neural metabolic pathways in mono- and co-cultures of primary rodent neural cells in 3D culture systems^55^,^56^,^57^.

Incubation with [1-^13^C]glucose revealed the presence of cell-specific metabolic features, in agreement with phenotypic data that showed heterogeneous differentiated neurospheres composed of neurons and astrocytes^15^. The observed amounts of C2 ^13^C labeled glutamate and glutamine indicate differential contributions of anaplerotic and oxidative pathways to pyruvate incorporation into the TCA cycle for the synthesis of these two metabolites. This is expressed as the PC/PDH ratio^48^, which was found to be 2.3-fold higher for glutamine synthesis, in comparison with glutamate. These observations are in agreement with previous reports in 2D primary cultures of murine cells and rat brains, showing the exclusive activity of PC in murine astrocytes^58^,^59^, the cellular compartment responsible for glutamine synthesis^60^. When analyzing the ^13^C-labeling in C4, similar amounts of [4-^13^C]glutamine and [4-^13^C]glutamate were observed, suggesting that astrocytes are present in higher numbers than neurons in these 3D cultures. Moreover, the higher contribution of the oxidative pathway for glutamate synthesis, relative to glutamine, and the presence of labeled GABA strongly suggest the presence of mature neuronal populations comprising glutamatergic and GABAergic lineages.

Incubation with [2-^13^C]acetate resulted in labeling patterns reflecting glial specific metabolism, in agreement with previous reports describing that acetate is mostly metabolized by astrocytes, in 2D murine primary cultures^37^,^38^. The overall percent enrichment in ^13^C labeling with [2-^13^C]acetate was similar to [1-^13^C]glucose incubation, demonstrating the importance of oxidative pathways for astrocytic metabolism. Our data demonstrate the presence of C2 labeled GABA, for which astrocyte-derived [4-^13^C]glutamine acts as precursor^37^. This indicates the establishment of such an important neuron-glia interaction as the glutamine-glutamate-GABA shuttle^32^. These findings, together with the observation of PC activity in astrocytes, reveal the presence of typical neural metabolic features important for neural tissue function. To our knowledge, this is the first report demonstrating these features in human stem-cell derived neural cells.

The presence of neuronal-astrocytic metabolic trafficking was further investigated by specifically challenging either astrocytic or neuronal populations, through MSO or acrylamide exposure, respectively. MSO has been widely described to act as an irreversible inhibitor of GS activity^61^, and has been linked to debilitating neurological disorders when ingested by animals or humans^62^. The presence of 20 μM MSO in differentiated neurospheres led to an almost complete inhibition of glutamine synthesis, as observed by the extensive reduction in the amount of ^13^C labeled glutamine. Also, the intracellular pools of BCAAs were found to be higher in MSO exposed cultures, suggesting a decreased catabolism of these amino acids. BCAAs have been described to have an important role in nitrogen turnover, contributing with their amino group to *de novo* glutamate biosynthesis, mainly through the activity of branched-chain aminotransferase (BCAT)^63^,^64^,^65^. Still, in the adult human brain and contrary to rodent neural cells, this enzyme is absent in astrocytes and is only expressed as a cytosolic isoform in neurons, mostly glutamatergic and GABAergic, and as a mitochondrial isoform in endothelial cells of the vasculature^66^. Our results suggest that MSO exposure can modulate BCAA metabolism in human neurons, where MSO can inhibit BCAT activity, although less potently than GS. This modulatory effect on BCAT had been proposed in studies on rodent brains^67^, but hadn’t been previously described for 2D neural cultures or in human neural cells. More importantly, MSO-treatment resulted in a significant decrease (50%) of GABA intracellular pool, likely due to shortage of glutamine from astrocytes. These results further support the establishment of the glutamine-glutamate-GABA cycle in the human cell model employed, and are in line with previous reports in 2D primary cultures of murine cells, arguing that GABAergic neurons are highly dependent on astrocyte-derived glutamine for neurotransmitter synthesis^37^.

Neurospheres were exposed to acrylamide to further challenge the neuronal-astrocytic metabolic interactions, in this case by targeting specifically the neuronal population. Acrylamide, a water-soluble alkene, causes cumulative neurotoxicity and neurodegeneration in adult humans and animals^68^. The mechanisms of acrylamide toxicity have been proposed to involve interference with kinesin-related motor proteins in neurofilaments, impairing axonal transport and ultimately leading to axonal degeneration and cell death^69^. More recently, the ability of acrylamide to form covalent adducts with highly nucleophilic cysteine thiolate groups located in active sites of presynaptic proteins has been suggested as the molecular basis for acrylamide-induced neuronal impairment^68^. Gene expression analysis on differentiated neurospheres exposed to 2.5 mM acrylamide confirmed the cell specificity of this compound, leading to significant negative modulation on expression levels of neuronal markers, while glial markers did not show any significant modulation. ^13^C-NMR spectroscopy data revealed significant alterations in the intracellular pools of ^13^C labeled glutamate and GABA, demonstrating the detrimental effects of acrylamide exposure on the neuronal populations. The amounts of labeled glutamate increased upon acrylamide exposure whereas labeling in GABA decreased. Although downregulation of GABA synthesis was likely to occur as outcome of the acrylamide-induced neurodegenerative processes, the increased glutamate synthesis was unexpected. One possible mechanism to explain glutamate accumulation is the impairment of synaptic activity in acrylamide-treated neurospheres, indicated by lower FM1-43 unloading kinetics. Acrylamide-induced neurotoxicity has been reported to affect synaptic vesicle trafficking and cycling, with observations of pre- and postsynaptic machinery structural^70^ and functional abnormalities, such as reduced number of presynaptic vesicles in the active zone, decreased neurotransmitter release and reduced neurotransmitter response^15^,^68^,^69^,^70^,^71^. These alterations are likely to impact the delicate neuronal-astrocytic contacts at synaptic sites, leading to impairment of metabolic interactions between these cell types. An imbalance in these interactions may also contribute to lower GABA synthesis upon acrylamide exposure, due to the high dependence of this neuronal sub-type on astrocyte-derived glutamine for GABA synthesis^37^. For astrocytes, a decrease in neurotransmitter release by neurons would represent a significant loss of carbon skeletons influx. Consequently, a continuous loss of TCA cycle intermediates to feed biosynthesis processes would occur in astrocytes, as for instance glutamine synthesis. In brain, the main enzyme responsible to replenish carbon skeletons loss due to exit of TCA cycle intermediates is PC^72^, and cells modulate its activity to supply the needs of *de novo* synthesis of these intermediates, as oxaloacetate^59^,^73^. This would explain our observation of an increase in [2-^13^C]glutamine (corrected for cycling, see Fig. 1), which derives from PC activity, in acrylamide-exposed neurospheres.

To our knowledge, this work demonstrates for the first time that *in vitro* differentiation of human neurons and astrocytes in a 3D setting allows the development of important and typical neural metabolic features found in the brain, such as the glutamine-glutamate-GABA cycle. Moreover, the combined data on acrylamide and MSO treatments showed that this human cell model and approach can be employed for toxicological studies, enabling the discrimination of the toxic effects on neuronal and astrocytic populations. The human 3D neural *in vitro* model employed can therefore contribute to a better understanding of human neural metabolism, namely neuron-astrocyte metabolic coupling, in pathological phenotypes that cannot be recapitulated in 2D cultures.

## Additional Information

**How to cite this article**: Simão, D. *et al*. Functional metabolic interactions of human neuron-astrocyte 3D *in vitro* networks. *Sci. Rep.* **6**, 33285; doi: 10.1038/srep33285 (2016).

## Figures and Tables

**Table 1:** Percentage of ^13^C enrichment for metabolites in cultures incubated with [1-^13^C]glucose or [2-^13^C]acetate.

		Percentage of ^13^C enrichment	
		[1-^13^C]glucose	[2-^13^C]acetate
Glutamine	**C2**	2.87 ± 0.64	3.98 ± 0.57
	**C3**	1.66 ± 0.39	2.52 ± 0.44
	**C4**	3.83 ± 0.72	7.98 ± 1.42
Glutamate	**C2**	5.18 ± 1.35	3.29 ± 0.55
	**C3**	3.74 ± 0.98	2.07 ± 0.13
	**C4**	9.85 ± 1.90	7.91 ± 0.36
Alanine	**C2**	1.59 ± 0.71	n.d.
	**C3**	15.88 ± 4.21	n.d.
Aspartate	**C2**	4.09 ± 1.24	2.83 ± 1.13
	**C3**	4.49 ± 1.34	3.88 ± 1.06
GABA	**C2**	11.08 ± 1.79	2.89 ± 0.59

Neurospheres were incubated with [1-^13^C]glucose or [2-^13^C]acetate and cell extracts were analyzed using ^13^C-NMRS. For more details, see Materials and Methods. Results are presented as mean ± s.e.m.
